# Individual, community and region level predictors of insecticide-treated net use among women in Uganda: a multilevel analysis

**DOI:** 10.1186/s12936-020-03412-4

**Published:** 2020-09-16

**Authors:** Edward Kwabena Ameyaw, Yusuf Olushola Kareem, Sanni Yaya

**Affiliations:** 1 School of Public Health, Faculty of Health, University of Technology Sydney, Sydney, NSW Australia; 2 Institute for Advanced Medical Research and Training, College of Medicine, University of Ibadan, Ibadan, Nigeria; 3 School of International Development and Global Studies, University of Ottawa, Ottawa, Canada; 4 The George Institute for Global Health, Imperial College London, London, UK

**Keywords:** Insecticide-treated net, ITN, Women, Malaria, Uganda, Public health, Global health, Malaria Indicator Survey

## Abstract

**Background:**

Use of insecticide-treated net (ITN) has been identified by the World Health Organization as an effective approach for malaria prevention. The government of Uganda has instituted measures to enhance ITN supply over the past decade, however, the country ranks third towards the global malaria burden. As a result, this study investigated how individual, community and region level factors affect ITN use among women of reproductive age in Uganda.

**Methods:**

The 2018–2019 Malaria Indicator Survey of Uganda involving 7798 women aged 15–49 was utilized. The descriptive summaries of ITN use were analysed by individual, community and region level factors. Based on the hierarchical nature of the data, four distinct binomial multilevel logistic regression models were fitted using the MLwiN 3.05 module in Stata. The parameters were estimated using the Markov Chain Monte Carlo (MCMC) estimation procedure and Bayesian Deviance Information Criterion was used to identify the model with a better fit.

**Results:**

The proportion of women who use ITN was 78.2% (n = 6097). Poor household wealth status [aOR = 1.66, Crl = 1.55–1.80], knowing that sleeping under ITN prevents malaria [aOR = 1.11, Crl = 1.05–1.24] and that destroying mosquito breeding sites can prevent malaria [aOR = 1.85, Crl = 1.75–1.98] were associated with higher odds of ITN use. ITN use attributable to regional and community level random effects was 39.1% and 45.2%, respectively.

**Conclusion:**

The study has illustrated that ITN policies and interventions in Uganda need to be sensitive to community and region level factors that affect usage. Also, strategies to enhance women’s knowledge on malaria prevention is indispensable in improving ITN use.

## Background

Malaria among women in the reproductive age has become a public health priority due to the enormous adverse implications it poses to their reproductive wellbeing [1]. Six countries account for more than half of the global malaria burden. All these countries are located in sub-Saharan Africa and includes Uganda where 5% of the global malaria cases occur [2]. Malaria is a public health priority linked with poverty, declined socio-economic development and is the commonly reported disease within both public and private health facilities in Uganda [3]. It is endemic in 95% of Uganda with the remaining 5% being irregular and epidemic-prone transmission areas [4]. Uganda accounts for the sixth highest annual malaria induced deaths in Africa with $0.41 to $3.88 malaria expenditure per person per month. One episode of malaria costs a family an average of 3% of their annual revenue. These include direct out-of-pocket expenses on consultation, drugs and transportation cost to distant health facilities [4].

Insecticide-treated net (ITN) has been identified by the World Health Organization (WHO) and the global community as an effective approach for malaria prevention. The government of Uganda has acknowledged this and instituted measures to enhance ITN supply to households. In the year 2000, the government waived importation taxes on ITNs and this was followed by establishment of the ITN Working Group of the inter-agency coordination committee for malaria (ICCM) in 2002. In 2009, the country scaled up to universal coverage of ITN (i.e. one ITN for every two persons). As of 2014, the National Malaria Control Program (NMCP) had distributed more than 12 million Long Lasting Insecticide Nets (LLNs) through mass campaigns and this culminated in 60% administrative coverage. The prime vision of the NMCP is to achieve “Malaria free Uganda” by providing quality reliable malaria prevention and treatment services to all persons. Women and children constitute the priority group of these interventions due to reduced immunity during pregnancy and immature immune system of children [1, 4].

Although, recent studies have revealed high ITN ownership in Uganda. However, ownership of ITN do not commensurate with utilisation. Nuwamanya et al. [5] reported 98.8% ownership of ITN with 91.1% of utilization. Although, this study was conducted after a mass ITN distribution exercise in Mbarara municipality. Similarly, another study in south western Uganda reported 84.0% of ownership of ITN among pregnant women, and only 66.1% reported consistent use [6]. In spite of the gap between ITN ownership and usage [5–7], little is known about the individual and contextual level factors associated with ITN use among women of reproductive age. Previously reported association between socio-demographics and ITN use have also been inconsistent in the literature. For example, a significantly positive association has been found between older age [8], higher education [9], and better household wealth [10] and ITN use, whereas some studies have shown that the association were insignificant or otherwise [11]. This study investigates individual, community and regional level factors associated with ITN use among women of reproductive age in Uganda.

## Methods

### Study design

The 2018–2019 Malaria Indicator Survey of Uganda (2018–19 UMIS) was utilized for this study. UMIS is a cross-sectional survey implemented by the Uganda Bureau of Statistics (UBOS) and National Malaria Control Division (NMCD) with technical support from the Inner City Fund (ICF) [12].

### Sampling technique

A two-stage sampling design was adopted in order to ensure the estimation of core indicators for three cardinal domains; national, rural/urban settings and the 15 regions of the country. Refugee settlements were captured as a distinct sampling domain. In the first phase of sampling, sample points (clusters) were selected from the sample frames, that is refugee and non-refugee settlements. The Enumeration Areas (EAs) earmarked for the 2014 National Population and Housing Census (NPHC) were used as the sampling frame. Three hundred and twenty (320) clusters were selected from the non-refugee settlement frame through probability proportional to size as defined by the NPHC. This emerged from rural (236) and urban (84) areas of the country. Within regions, urban settings were oversampled in order to generate unbiased estimations for urban domains. For refugee frame, 22 clusters were selected following the same procedure. The second phase constituted a systematic selection of households by listing households and eligible respondents were randomly selected thereafter. A total of 28 households were selected from each EA yielding a total sample size of 8878 households. All women aged 15–49 who were either permanent residents of selected households or visitors who joined the households the night before the survey were eligible for the survey. Within selected households, 8389 women were eligible for interview and 8231 were interviewed successfully culminating in 98% response rate for the non-refugee settlements while the refugee settlement, had 99% response rate [12].

### Inclusion criteria and sample size

This study included women aged 15–49 who indicated that they possessed ITN during the 2018–2019 Malaria Indicator survey. Based on this, a total of 7798 women were included in the analysis.

### Data collection

The data collection exercise occurred between December 11, 2018 and January 31, 2019. Twenty-three teams were formed for the data collection and each of these had seven members. Information about these women were gathered with three questionnaires. These are the household questionnaire, woman’s questionnaire and the biomarker questionnaire. To mitigate possible language barrier and ensure clarity, the questionnaires were translated from English Language to the local languages namely Luganda, Luo, Lugbara, Ateso, Runyankole/Rukiga, and Runyoro/Rutoro. Whilst the household and woman’s questionnaires were programmed to tablets to permit computer-assisted personal interviewing (CAPI), the biomarker questionnaire was in hard copy and subsequently entered to the CAPI platform after completion. Listing of usual members of households and visitors, gathering of basic information on each listed person and data on characteristics of the households' dwelling unit were done with the household questionnaire. The basic information comprised age, sex and relationship to the household head. Characteristics of household dwelling unit included toilet facilities, indoor residual spraying, and ownership and use of mosquito nets. The woman’s questionnaire was used for collecting information from all women aged 15–49 whilst the biomarker questionnaire was used to capture the results of the anaemia and malaria testing of the children 0–59 months.

### Outcome variable

ITN use was the outcome variable for the study. Those who indicated that they slept under the treated mosquito net the night before the survey were considered as using ITN (coded as 1) whereas those who did not sleep under net were considered as not using ITN (coded as 0).

### Explanatory variables

#### Individual level factors

Eight variables were considered at the individual level; age, education, household wealth index, pregnancy status, if mosquito bite causes malaria, if sleeping under ITN prevents malaria, if destroying mosquito breeding sites prevent malaria and household size. Age was categorized as 15–19, 20–24, 25–29, 30–34, 35–39, 40–44 and 45–49. Education was measured as highest level of educational attainment and categorized into no education, primary, secondary and higher. The household wealth index was derived from aggregation of household’s asset possession. Thus, items such as type of floor/roofing, radio, television, car, toilet and source of water were computed into wealth index through principal component analysis and categorized into poorest, poorer, middle, richer and richest. These were re-categorized into poor middle and rich. Pregnancy status was classified as pregnant, no or not sure. Household size was categorized into less than five and five or more. These three variables: if mosquito bite causes malaria, if sleeping under ITN prevents malaria and if destroying mosquito breeding sites prevents malaria, had binary responses (yes or no).

#### Community level factors

Three variables were considered at the community level. The first was residence categorized as urban, rural and refugee settlements. The second variable was socio-economic status computed using principal component analysis and comprised proportion of women who were poor and uneducated within communities. The socioeconomic status was categorized into tertiles: tertile 1 (least disadvantaged), tertile 2 and tertile 3 (most disadvantaged). The third variable was region categorized into Eastern, Northern, Western and Southern Uganda.

#### Region level factors

Socio-economic status at the regional level was derived by using principal component analysis to compute the proportion of women who were poor and uneducated within the regions and then categorized into tertiles: tertile 1 (least disadvantaged), tertile 2 and tertile 3 (most disadvantaged).

### Statistical analysis

The descriptive summaries of the individual, community and regional level factors by ITN utilization were computed. A Chi squared test of association was performed for each of the explanatory variable and ITN use. Four distinct multilevel binary logistic regression models with hierarchical structure were fitted thereafter. The first (null) model had no explanatory variable. The null model decomposed the magnitude of variation in ITN use that can be explained by community and regional random effects. The second model then included the individual-level variables, the third model comprised both individual and community level variables while the fourth (full) model, controlled for individual, community and region level factors. The fixed effects of all the models were computed using odds ratios (ORs) with corresponding 95% credible intervals (Crls) whereas the random effects were measured with intra-cluster correlation coefficient (ICC) and median odds ratio (MOR) [13, 14].

#### Model fit and specification

Multicollinearity was checked using variance inflation factor (VIF) with greater than 5 as cut-off [15]. The parameters were estimated using the Markov Chain Monte Carlo (MCMC) [16] approach. In order to fit the most parsimonious model, the Bayesian Deviance Information Criterion (DIC) was used as a test for model fit, a model with a lower value of DIC has a better fit. All the fitted models were analysed using the MLwiN command version 3.05 [17] in the Stata software version 13 (StataCorp, College Station, TX).

### Ethics approval

Protocol for the 2018–2019 UMIS was approved by the Uganda National Council for Science and Technology (UNCST), the Ethics Committee of the School of Medicine Research and Ethics Committee (SOMREC) of the Makerere University and the institutional review board at the ICF. All participants consented before participating in the survey and the data were anonymized at the data finalization stage.

## Results

### Descriptive results

Table 1 presents ITN use among Ugandan women by socio-demographic characteristics at the individual, community and region level. A total of 6097 women (78.2%) used ITN. A higher proportion of women age 45–49 (85.8%) and with tertiary education (83.4%) utilized ITN. Most women who knew that mosquito bite causes malaria reported ITN use (79.6%). A higher proportion of those who indicated that sleeping under mosquito net prevents malaria utilized ITN (78.6%) and similarly among women who indicated that destroying mosquito breeding sites can prevent malaria (79.5%). Most women in households with 5 or more members indicated that they utilized ITN (85.1%). At the community level, a higher proportion of women (88.1%) who resided in refugee settlements, most disadvantaged communities (79.8%) and in Southern Uganda (81.3%) reported ITN use. At the regional level, a higher proportion of women in a moderately disadvantaged region (78.6%) utilized ITN compared to women in the least and most disadvantage regions.

**Table 1:** ITN use at individual, community and region level independent variables. (Source: 2018–19 Uganda Malaria Indicator Survey)

	ITN UTILISATION			*p* value
	No	Yes	Total	
	n (%)	n (%)	n (%)	
Individual level				
Age				< 0.001
15–19	600 (33.4)	1196 (66.6)	1796 (100)	
20–24	329 (22.1)	1153 (77.8)	1481 (100)	
25–29	244 (18.9)	1048 (81.1)	1292 (100)	
30–34	185 (16.5)	934 (83.5)	1119 (100)	
35–39	147 (16.3)	755 (83.7)	902 (100)	
40–44	128 (17.7)	595 (78.3)	724 (100)	
45–49	69 (14.2)	414 (85.8)	483 (100)	
Education				< 0.01
No education	191 (19.1)	806 (80.9)	997 (100)	
Primary	920 (22.7)	3131 (77.3)	4051 (100)	
Secondary	501 (22.6)	1712 (77.4)	2213 (100)	
Higher	89 (16.6)	448 (83.4)	537 (100)	
Household wealth index				< 0.01
Poor	400 (21.3)	1478 (78.7)	1878 (100)	
Middle	621 (22.9)	2087 (77.1)	2709 (100)	
Rich	680 (21.2)	2531 (74.0)	3211 (100)	
Pregnancy status				< 0.01
No or not sure	1569 (21.8)	5616 (78.2)	7185 (100)	
Pregnant	132 (21.5)	481 (78.5)	613 (100)	
Mosquito bite causes malaria				0.065
No	1124 (22.6)	3850 (77.4)	4974 (100)	
Yes	577 (20.4)	2247 (79.6)	2824 (100)	
Sleeping under mosquito net prevents malaria				< 0.001
No	443 (23.0)	1486 (77.0)	1929 (100)	
Yes	1258 (21.4)	4611 (78.6)	5869 (100)	
Destroying mosquito breeding site can prevent malaria				0.828
No	1369 (22.2)	4804 (77.8)	6173 (100)	
Yes	332 (20.5)	1292 (79.5)	1625 (100)	
Household size				< 0.001
Less than 5	1391 (24.4)	4321 (75.6)	5712 (100)	
5 or more	310 (14.9)	1775 (85.1)	2085 (100)	
Community level factors				
Residence				< 0.001
Urban	410 (19.2)	1724 (80.8)	2135 (100)	
Rural	1229 (23.9)	3914 (76.1)	5143 (100)	
Refugee settlements	62 (11.9)	458 (88.1)	520 (100)	
Socio-economic disadvantage				< 0.01
Tertile 1 (least disadvantaged)	655 (20.9)	2472 (79.1)	3127 (100)	
Tertile 2	692 (23.8)	2222 (76.2)	2914 (100)	
Tertile 3 (most disadvantaged)	354 (20.2)	1403 (79.8)	1757 (100)	
Region				< 0.001
Eastern	642 (24.2)	2005 (75.8)	2647 (100)	
Northern	415 (22.5)	1431 (77.5)	1846 (100)	
Western	298 (20.4)	1162 (79.6)	1460 (100)	
Southern	346 (18.7)	1499 (81.3)	1845 (100)	
Region level factors				< 0.001
Socio-economic disadvantage				
Tertile 1 (least disadvantage)	947 (23.0)	3164 (77.0)	411 (100)	
Tertile 2	420 (20.0)	1707 (80.0)	2127 (100)	
Tertile 3 (most disadvantaged)	334 (21.4)	1226 (78.6)	1560 (100)	
N	1701 (21.8)	6097 (78.2)	7798 (100)	

### Measures of association (fixed effects)

The results for the fitted models were presented in Table 2. The multicollinearity test indicated that there was no collinearity between the independent variables [highest VIF = 3.71, lowest VIF = 1.72, mean VIF = 2.08]. The final model consists of individual, community and region level variables. Household size of five or more, poor household wealth status, knowledge on whether sleeping under mosquito net prevents malaria and whether destroying mosquito-breeding site can prevent malaria were positively associated with ITN use. Women in poor household wealth quintile [aOR = 1.66, Crl = 1.55–1.80] had higher odds of ITN utilisation compared to those in the rich quintile. Women who perceive that sleeping under ITN prevents malaria had a higher odds of ITN use [aOR = 1.11, Crl = 1.05–1.24]. Similarly, women who believed that destroying mosquito breeding sites can prevent malaria had higher odds of ITN use compared to those who did not think so [aOR = 1.85, Crl = 1.75–1.98]. Women whose household members were less than five were associated with lower odds of ITN use [aOR = 0.66, Crl = 0.58–0.74]. Also, rural residents were less likely to use ITN [aOR = 0.63, Crl = 0.41–0.98].

**Table 2:** Multilevel logistic regression of individual, community and region level correlates of ITN use (Source: 2018–19 Uganda Malaria Indicator Survey)

	Model 1	Model 2	Model 3	Model 4
		OR [95% CrI]	OR [95% CrI]	aOR [95% CrI]
Fixed effect				
Individual level				
Age				
15–19		*0.40[0.32*–0.50]***	*0.41[0.33*–0.51]**	*0.39[0.31*–0.49]***
20–24		*0.67[0.54*–0.83]***	*0.70[0.56*–0.87]**	*0.66[0.51*–0.84]***
25–29		0.87[0.69–1.09]	0.90[0.72–1.13]	0.86[0.67–1.10]
30–34		1.08[0.86–1.36]	1.13[0.90–1.42]	1.07[0.83–1.38]
35–39		1.07[0.84–1.35]	1.10[0.86–1.41]	1.05[0.80–1.37]
40–44		0.96[0.74–1.24]	0.99[0.76–1.28]	0.94[0.72–1.23]
45–49		Ref	Ref	Ref
Education				
No education		*0.70[0.52*–0.94]**	*0.77*[*0.58*–*1.01*]	*0.72[0.54*–0.96]*
Primary		0.86[0.66–1.11]	0.93[0.72–1.19]	0.86[0.68–1.10]
Secondary		0.92[0.71–1.18]	0.99[0.78–1.26]	0.93[0.73–1.18]
Higher		Ref	Ref	Ref
Household wealth index				
Poor		*1.64[1.54*–1.76]***	*1.65[1.54*–1.78]***	*1.66[1.55*–1.80]**
Middle		*1.83[1.71*–1.97]**	*1.83[1.71*–1.96]**	*1.84[1.72*–1.98]***
Rich		Ref	Ref	Ref
Pregnancy status				
No or not sure		0.93[0.78–1.11]	0.97[0.81–1.15]	0.94[0.79–1.12]
Pregnant		Ref	Ref	Ref
Mosquito bite causes malaria				
No		0.90[*0.81*–*1.01*]	0.91[*0.82*–*1.01*]	0.90[*0.81*–*1.00*]
Yes		Ref	Ref	Ref
Sleeping under mosquito net prevents malaria				
No		Ref	Ref	Ref
Yes		1.12[0.99–1.26]	1.13[1.01–1.26]*	1.11[1.05–1.24]**
Destroying mosquito breeding site can prevent malaria				
No		*0.87[0.76*–0.99]*	*0.87[0.75*–0.99]*	*1.85[1.75*–1.98]*
Yes		Ref	Ref	Ref
Household size				
Less than 5		*0.66[0.59*–0.74]***	*0.66[0.59*–0.75]**	*0.66[0.58*–0.74]***
5 or more		Ref	Ref	Ref
Community level factors				
Residence				
Urban		0.87[0.59–1.29]	0.93[0.55–1.56]	0.72[0.44–1.18]
Rural		0.76[0.54–1.06]	0.77[0.49–1.22]	*0.63[0.41*–0.98]*
Refugee settlements		Ref	Ref	Ref
Socio-economic disadvantage				
Tertile 1 (least disadvantaged)			Ref	Ref
Tertile 2			1.04[0.84–1.29]	1.02[0.82–1.26]
Tertile 3 (most disadvantaged)			0.87[0.66–1.16]	0.89[0.67–1.18]
Region				
Eastern			1.71[0.94–3.11]	1.30[0.70–2.42]
Northern			*2.20[1.15*–*4.24]*	*5.08[1.95*–13.20]***
Western			2.22[0.99–4.95]	1.35[0.69–2.63]
Southern			Ref	Ref
Region level factors				
Socio-economic disadvantage				
Tertile 1 (least disadvantage)				Ref
Tertile 2				1.26[0.79–1.99]
Tertile 3 (most disadvantaged)				*0.33[0.14*–0.74]***
Measures of variation				
Region level				
Variance (SE)	2.342[1.869–2.936]	0.168[0.061–0.391]	0.181[0.050–0.469]	0.052[0.003–0.175]
ICC (%)	39.1[35.7–44.2]	32[27.8–36.1]	37.45[32.74–43.27]	46.29[42.13–52.62]
MOR	4.30[3.68–5.13]	4.19[3.82–4.53]	3.98[3.22–4.31]	4.28[3.99–4.58]
Explained variation (%)	Reference	52.86[48.41–59.34]	48.72[42.74–54.79]	68.11[63.41–72.63]
Community level				
Variance (SE)	0.349[0.068–0.414]	0.344[0.259–0.448]	0.352[0.269–0.450]	0.352[0.270–0.447]
ICC (%)	45.20[37.0–50.4]	37.62[31.39–42.85]	44.21[38.74–47.31]	48.41[40.55–52.17]
MOR	1.76[1.28–1.85]	2.38[2.04–3.02]	3.54[3.12–4.11]	3.57[3.13––4.11]
Explained variation (%)	Reference	41.62[38.43–50.8]	39.51[33.59–43.29]	40.32[37.66–46.23]
Model fit statistics				
Bayesian DIC	10,345.46	10,073.41	10,073.12	10,072.31
N	7798	7798	7798	7798

*DIC* Deviation information criterion, *CrI* credible interval, *ICC* intra-cluster correlation, *MOR* median odds ratio, *SE* standard error

^*^ *p* < 0.05, ^**^ *p* < 0.01, ^***^ *p* < 0.001

### Measures of variation (random effects)

The results of the random effects are also presented in Table 2. Outcome of the first model (empty) indicated that substantial variation in ITN use exist across regions [σ^2^ = 2.34, 95% Crl = 1.87–2.94]. The intra-region correlation coefficient shows that 39.10% [Crl = 35.7–44.2] of the variance in the likelihood of ITN use is attributable to region level factors. Also, 45.2% odds [Crl = 37.0–50.4] of ITN use is attributable to community level factors. From model 4, the MOR reveals that when a woman relocates to a different region with higher likelihood of ITN use, the odds of ITN utilisation increases by 4.28. Similarly, the odds of ITN utilization increases by 3.57 if a woman moves to a community with a higher likelihood of ITN use.

## Discussion

This study aimed at investigating how individual, community and region level factors affect ITN use among women aged 15–49 in Uganda. The findings indicate that indeed factors at these three levels affect ITN use in the country. This suggests that ITN use transcends personal level characteristics and highlights the importance of community and region level factors. It therefore calls for a hierarchical intervention that considers the dynamics of ITN use and malaria prevalence at the regional, community and household levels. Previous studies have indicated discomforts such as heat, scent, net washing, vomiting and difficulty in hanging the net among other factors interfere usage [18–21]. In the case of Uganda, this current study draws attention to the need to investigate which of these arrays of factors attenuate ITN use in the respective communities and regions so as to revise current interventions accordingly to render them more relevant and responsive.

Poor women were more likely to use ITN. This outcome resonates with evidence from Rwanda and Ghana [22, 23]. Through the National Malaria Control Programme, the President’s Malaria Initiative and other anti-malaria interventions, the government of Uganda and other stakeholders have ensured easy access to ITN and other malaria control measures [3, 4]. For instance, over 7.2 million LLINs were distributed in 2010 under the National Malaria Control Programme and as of 2013/2014, about 12 million LLINs had been distributed through mass campaigns where 60% administrative coverage was achieved with the aim of achieving the “Malaria free Uganda” vision [4]. These interventions are predominantly pro-poor because the rich are more likely to reside in less malaria prone environments unlike the poor who may be in environments with poor drainage system and unkempt environments where mosquito breeding sites are immanent [22, 24]. On the account of these, the poor are likely to embrace and effectively use ITNs. Due to the economic advantageous position of the rich, they can explore other malaria prevention strategies such as indoor residual spraying (IRS) or use of mosquito repellents with ease [24, 25]. Conversely, these options may be “luxurious” to the poor women and hence effective utilisation of readily available ITN. The finding indicates that ITN distribution, advocacy interventions and policies need to target poor women in Uganda because they may have a critical demand for ITNs relative to the rich.

Women who perceived that sleeping under ITN prevents malaria were more likely to sleep under ITN. Similarly, women with the perception that destroying mosquito breeding site can prevent malaria had higher odds of using ITN. Undoubtedly, women who know these preventive strategies are more knowledgeable about the causes of malaria and its prevention [26]. These findings reflect the proposition of the health belief model [27]. Strecher and Rosenstock [27] espoused that one’s ability to initiative a healthy or protective intervention or lifestyle is not only a function of the person’s characteristics but also the wealth of knowledge and information held about the phenomenon. On this score, high ITN use is expected among women who believe that sleeping under ITN and destroying mosquito-breeding sites can prevent malaria. Our observed results concur with reports from South Eastern Asia [18, 28, 29]. The findings prompt a compelling need to intensity ITN education and mass media campaigns on the importance of ITN use and risks associated with doing otherwise in the absence of other preventive measures. Such education and campaigns can have wider coverage and higher impact when channelled through widely utilized mass media platforms such as radio, which is noted to be the most vibrant and commonest medium for public discussion and ITN advertisements in Uganda [30, 31].

Further, women in households with less than five members were less likely to use ITN. This finding in inconsistent with the observation made by Nkoka et al. [32] in Malawi where ITN use was more probable for women from households with less persons. This finding is, however, consistent with a Cameroon-based study on coverage and usage of insecticide-treated nets (ITNs) within households. Unlike smaller households, households with greater or more members are more likely to have someone who can educate other household members on the relevance of ITN [33].

Women in rural locations also had less likelihood of ITN use. This may reflect inequity in ITN outlets in favour of urban locations or limited education in rural settings. In contrast, Bennett et al. [34] noted that rural residents in Cameroon stand a higher chance of using ITN compared with urban residents following a national mass distribution exercise. Another study from Ghana revealed that rural residents are more likely to use ITN [22]. Contextual variations between Uganda and settings for the other studies may account for the inconsistent findings. Scrutiny of rural/urban distribution of ITN and advocacy strategies may be required to enhance ITN use in rural Uganda.

### Strengths and limitations

This study is supported by data from the most recent malaria survey of Uganda and, therefore, presents current estimates. The study uses large sample size, which renders the findings generalizable to Uganda women. The study offers information on ITN use taking cognisance of community and region level factors. In spite of these strengths, the study is not devoid of limitations. First, depending on a woman’s community, there is the possibility of social desirability bias about ITN use. Secondly, this is a cross-sectional study and as such causal inference in not possible.

## Conclusions

The study has indicated that community and region level factors influence ITN use in Uganda. Being poor and possession of knowledge about malaria prevention increased prospects of ITN use. There is, therefore, a need to review the existing ITN inventions and revise taking cognisance of community, and regional level variations. Moreover, the socio-economic status of women and depth of knowledge ITN are critical factors worth considering in planning. Future study may focus on rural–urban variation in ITN use in Uganda.

## Data Availability

The dataset supporting the conclusions of this article is available in the Measure DHS repository, [https://www.malariasurveys.org].

## Abbreviations

aOR: Adjusted Odds Ratio
CAPI: Computer-assisted personal interviewing
Crl: Credible interval
DIC: Deviance Information Criterion
EAs: Enumeration areas
ICCM: Inter-agency coordination committee for malaria
ICF: Inner City Fund
ICC: Intra-cluster correlation coefficient
ITN: Insecticide-treated net
LLIINs: Long Lasting Insecticide Nets
MCMC: Markov Chain Monte Carlo
MOR: Median odds ratio
NPHC: National Population and Housing Census
NMCD: National Malaria Control Division
NMCP: National Malaria Control Programme
SOMREC: School of Medicine Research and Ethics Committee
UMIS: Uganda Malaria Indicator Survey of Uganda
UNCST: Uganda National Council for Science and Technology
VIF: Variance inflation factor
WHO: World Health Organization
